# Prevalence of Smoking and Beliefs and Attitude Toward Smoking Habit and Smoking Cessation Methods Among Pharmacy Students: A Cross-Sectional Study in Saudi Arabia

**DOI:** 10.3389/fpubh.2022.816101

**Published:** 2022-04-01

**Authors:** Abdulrahman Alwhaibi, Syed Wajid, Ahmad Alenezi, Yazeed Salami, Ibrahim Alhaydan, Sana Samreen, Abdulaziz Alhossan, Mohamed N. Al-Arifi

**Affiliations:** Department of Clinical Pharmacy, King Saud University, Riyadh, Saudi Arabia

**Keywords:** smoking, attitudes, prevalence, anti-smoking, Saudi Arabia

## Abstract

**Objective:**

The impact of pharmaceutical services on public health especially in regards to smoking cessation counseling can influence the rate of smoking cessation. The present study aims to evaluate prevalence, beliefs, and attitude of pharmacy students toward smoking habit and SC methods.

**Methods:**

An online cross-sectional survey was conducted among pharmacy students at King Saud University, Riyadh, Saudi Arabia over 4-month period from May to August 2021. The survey consisted of 22-items focused on evaluating the prevalence, belief, and attitude toward smoking habits and smoking cessation methods. Data was descriptively analyzed using a statistical package for social science version 26 (SPSS).

**Results:**

A total of 675 students completed the survey, among which 78.7 % were non-smokers and only 31.7% received education on the dangers of smoking. The most common methods of smoking cessation they heard of were consultation (58.6%), followed by use of electronic cigarettes (41.92 %) and Nicotine patch (40.7%). One-third of the surveyed students (36.8 %) agreed that specialized smoking cessation clinics enhance the efficacy of smoking cessation methods. The majority of students (80.6%) agreed to ban smoking in public places and 92.2% believed that tobacco sales to adolescents should be forbidden. Health professionals should actively participate and advise their patients to quit smoking as 89.8 % students agreed on that. Age and gender of students had significantly influenced the prevalence of smoking, history of smoking, frequency of smoking, efforts to quit smoking among smokers (*p* = 0.0001).

**Conclusion:**

The prevalence of non-smokers among pharmacy students is encouraging, yet level of awareness about and usefulness of smoking cessation methods is unsatisfactory. Therefore, the study clearly highlights a great need for integrating smoking cessation programs in their academic curricula to prepare them for real-world practice.

## Introduction

Smoking is one of the major health risk factors, which reduces not only an individual's physical performance but also quality of life (1). In Saudi Arabia, studies indicated that the number of smoking-related fatalities are on the rise, with an estimated 70, 000 Saudis dying each year from smoking-related illnesses (2, 3). Regionally and globally, the prevalence of smoking has increased these days and become a major public health problem that is believed to continue growing (4). Likewise, the World Health Organization (WHO), the Centers for Disease Control and Prevention (CDC), and partners initiated the Global Tobacco Surveillance System (GTSS) and promoted smoking cessation (SC) programs and treatment using evidence-based tobacco cessation guidelines (5).

Smoking cessation has gained more popularity due to its safety and effectiveness in reducing the risk of smoking-related diseases such as cancer, cardiovascular diseases, and respiratory diseases (6). SC treatments consist of nicotine replacement therapy (NRT) and non-nicotine replacement therapy (Non-NRT), including bupropion and varenicline (7–10). Studies suggested that a dramatic decline in fatality rate attributed to smoking-related cardiovascular and lung diseases occurs if smokers quit smoking at any point in their life (11, 12), hence smoking cessation effort should be supported by health care professionals to achieve early and long-term healthy life. Health care students should learn, and educate public about the dangers of smoking and advocate and guide, as future professionals, patients who smoke to quit smoking either by advising or referring them to SC clinics (13, 14).

There is a wealth of studies on smoking and smoking cessation published from Saudi Arabia (15–17). According to a recent study, young adults are less likely to use SC interventions compared to older adults (18, 19) and approximately one out of ten people seek or obtain evidence-based treatments (18–21). Previous literature demonstrated that health care students have inadequate knowledge and receive very little or no education on SC techniques (22, 23). Yet in many developing countries, no SC education is provided at schools or colleges levels (24).

The role of a pharmacists in society is unique since they can initiate alterations in smoking habits in their communities, as there is no appointment required to meet them as well as being the most accessible healthcare provider to the public with no cost (25). Additionally, pharmacists have a broad relationship to patients and are considered a trustworthy source of health-related information, so they can provide numerous pharmacological SC interventions, such as nicotine replacement formulations that can be purchased from community pharmacies without prescription. Moreover, they can advise and guide smokers after receiving proper and specific training or education on SC strategies (25). For the successful development of tobacco control measures, curricula in pharmacy colleges must include teaching modules to students focusing on the responsibility toward this matter and educating on SC methods and techniques. Overall, the knowledge and beliefs about smoking and smoking cessation among pharmacy students is crucial. Despite the wealth of literature about smoking behaviors in colleges' students (15–17), there is a scarcity of research on the prevalence, beliefs, and attitude of pharmacy students toward SC methods. The present study aims to evaluate prevalence, beliefs, and attitude of pharmacy students toward smoking habit and SC methods.

## Methods

A cross-sectional online questionnaire-based study was conducted between May to August 2021 among King Saud University Pharmacy Students, Riyadh, Saudi Arabia. Targeted population included pharmacy students aged ≥18 years, speaking Arabic or English and willing to participate in the study by singing the consent. The participants were assured that the data would be used only for the purpose of research and would be maintained confidential throughout the study. Others who do not match the inclusion criteria were excluded. The study was approved by the institutional review board of King Saud University College of Medicine with the following reference number: E-21-6430.

The questionnaire was designed using a previously published study with similar objectives (15, 16). The questionnaire was divided into two sections. The first section included 11 items that assessed participants' characteristics like age, gender and certain information about smoking habits such as history of smoking, frequency of smoking, education on dangers of smoking, methods used to quit smoking. The second section contained 10 items regarding beliefs (4-items) and attitude toward usefulness of smoking cessations methods (4-items) and knowledge about smoking cessations (2- items). The beliefs section was assessed using five-point Likert scale (strongly agree to strongly disagree), while attitude was assessed on four-point scale (very useful to I don't know). The last two items (knowledge) were collected using multiple choice options. The questionnaire was evaluated by a research team, to ensure the readability and accuracy of the content. Later, the final questionnaire version was translated into Arabic by a certified Arabic speaker. The designed Arabic version was subjected to, a pilot study among randomly selected sample of 10 participants to give their opinions on simplifying the questionnaire, if needed. Reliability test was done and Cronbach's alpha value of 0.65 was obtained indicating that the questionnaire was useful to carry out the study.

The final study questionnaire was distributed online through the social media applications such as WhatsApp, Twitter, and Facebook. Other methods to improve the response rate such as emails, were considered. An invitation link containing a survey questionnaire was sent to the participants. To avoid response duplications, participants were instructed to fill the questionnaire once despite the method through which they received it. For the data collection, we used the snowball technique where any person recruited to fill the questionnaire provides multiple referrals.

## Data Analysis

The data were analyzed using the Statistical Package for the Social Sciences (SPSS) (version 26 for Windows (SPSS Inc., Chicago, Illinois)). Descriptive statistic was used to summarize the demographic characteristics. Univariate analyses (Chi-squared test, / Fishers exact test) were used to test the difference in the variables as appropriate. *P* < 0.05 and a 95 % confidence interval (CI) indicated significant results.

## Results

A total of 675 students completed the questionnaire, of which 50.8% (n=343) were males and 49.2% (n=332) were females, as shown in Table 1. The 18 to < 23 year's age group was predominant with 71.7 %, followed by the 23 to < 25 year's age group with 22.2 %. The prevalence of smoking among all students was 20.3% (*n* = 137), among which daily smokers accounted for 81.29% (*n* = 110). Only 31.7% (*n* = 214) received education on the dangers of smoking. Regarding quit smoking, approximately 69.34% (*n* = 95) of the students have tried. Detailed information on the demographic characteristics and smoking status of the respondents were shown in Table 1.

**Table 1:** Demographic characteristics and smoking behavior among 675 students.

**Parameters**	**Frequency (*n*)**	**Percentage** **(%)**
**Gender**		
Male	343	50.8
Female	332	49.2
**Age (full years)**		
18– <23	484	71.7
23– <26	150	22.2
26– <30	25	3.7
At least 30	16	2.4
**Do any of your parent's work in a health care setting?**		
Yes	627	92.9
No	48	7.1
**What is your nationality?**		
Saudi	640	94.8
Non-Saudi	35	5.2
**Do you smoke?**		
Yes	137	20.3
No	538	79.7
**How many cigarettes do you smoke per day (** ***n*** )? ^*^		
<10	38	27.74
10–20	68	49.63
>20	13	9.48
I don't smoke daily	18	13.13
How long have you been smoking?^*^		
<1 Year	8	5.84
1–3 years	33	24.08
>3 years	96	70.07
How frequent are you smoking? ^*^		
Daily smoker	110	81.29
Occasional smoker	26	18.97
Never smoker	1	0.73
Have you tried to quit smoking? ^*^		
Yes, more often	28	20.43
Yes, sometimes	67	48.91
No, I never tried	26	18.97
I don't want to quit smoking	15	10.95
**Have you received any education on the dangers of smoking?**		
Yes	214	31.7
No	276	40.9
I don't know	185	27.4

* *n = 137*.

**Figure 1:**
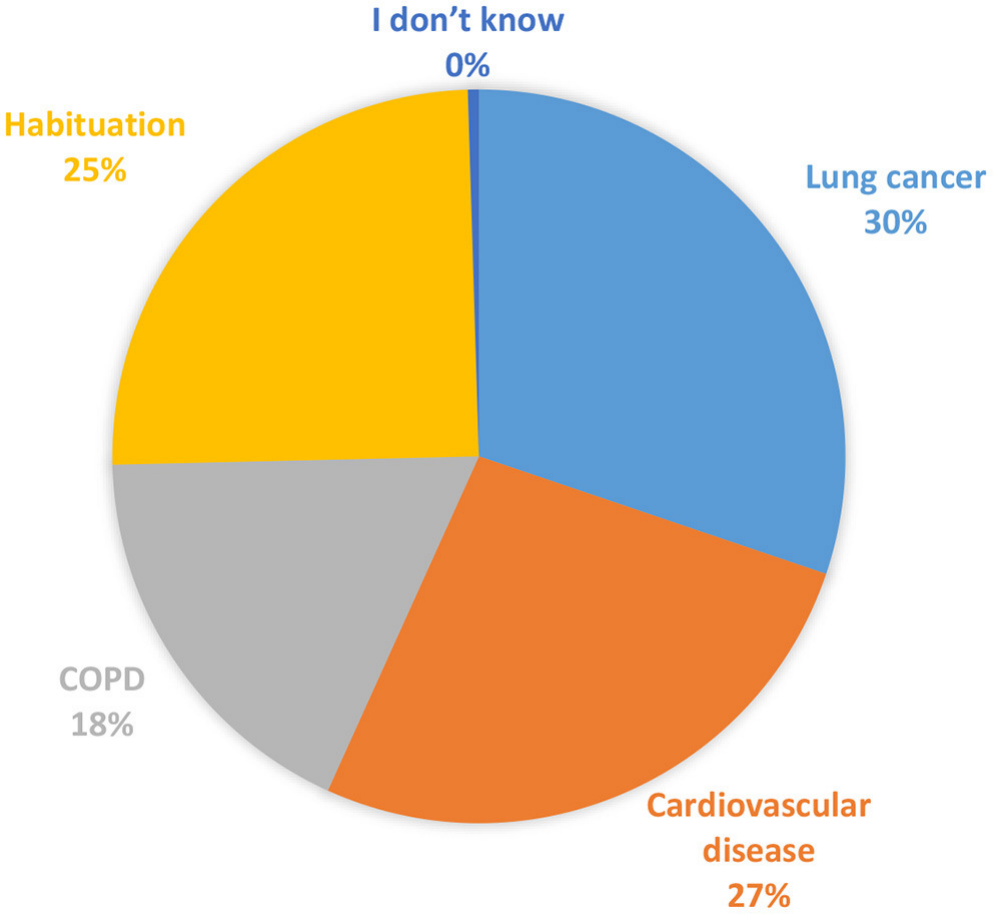
The most important hazards faced by the smokers.

Slightly more than half of the students (58.6%) believed that consultation is the SC method they had heard before, followed by electronic cigarettes (ECs) (41.92%), nicotine patch (40.7%), and nicotine gum (27. 1%), as shown in Table 2. When smokers were asked about the SC methods they had tried before, about 9.5% reported ECs while never tried by 17.33%. According to smokers' beliefs, the most important hazards they face are lung cancer 125 (30%), followed by cardiovascular disease 110 (27%) and habituation 103 (25%), as demonstrated in Figure 1.

**Table 2:** Student's knowledge toward smoking cessation methods (*n* = 675).

**Items**	**Frequency (*n*)**	**Percentage (%)**
Which method(s) of SC have you heard before? ^*^(Select all that apply)		
Consultation	396	58.6
Electronic cigarettes	283	41.92
Nicotine Patch	275	40.7
Nicotine Gum	183	27.1
I don't know	151	22.37
Nicotine Spray	61	09
Bupropion(Zyban)	34	5.03
Acupuncture	33	4.8
Which method(s) of SC have you tried before? ^*^(Select all that apply) ^*^		
Nicotine Spray	2	0.296
Consultation	15	2.22
Nicotine Patch	16	2.37
Nicotine Gum	7	1.03
Acupuncture	1	0.148
Bupropion(Zyban)	1	0.148
Electronic cigarettes	64	9.48
I don't try	117	17.33

* *n = (137)*.

Regarding students' beliefs toward SC, approximately 80.6% agreed to ban smoking in public places, as shown in Table 3. The majority of the students (92.2%) and 75.4% believed that tobacco sales to adolescents and tobacco smoking in universities and cafes should be forbidden, respectively. Detailed information is presented in Table 3.

**Table 3:** Beliefs of students toward smoking cessation (*n* = 675).

**Items**	**Strongly agree *n* (%)**	**Agree *n* (%)**	**Don't know *n* (%)**	**Disagree n (%)**	**Strongly disagree n (%)**
Smoking in public places should be banned	474 (70.2)	70 (10.4)	50 (7.4)	59 (8.7)	22 (3.3)
Tobacco sales to adolescents should be forbidden	569 (84.3)	53 (7.9)	27 (4.0)	10 (1.5)	16 (2.4)
Tobacco smoking in universities and cafes should be forbidden	460(68.1)	49(7.3)	58(8.6)	68(10.1)	40(5.9)
Health professionals should advise their patients to quit smoking	480 (71.1)	126 (18.7)	54 (8.0)	7 (1.0)	8 (1.2)

Approximately one-third of the students (36.8 %), 23.1, 22 and 26.3% believed that specialized smoking cessation clinics, university hospitals, on-campus clinics and family physicians visits are useful and effective smoking cessations methods. However, the majority of the students don't know about specialized smoking cessation clinics (53.2%). Detailed descriptions of the responses are shown in Table 4.

**Table 4:** Evaluating the usefulness of smoking cessations methods (*n* = 675).

**Items**	**Very *n* (%)**	**Useful**	**Not *n* (%)**	**know *n* (%)**
	**useful *n* (%)**	***n* (%)**	**useful *n* (%)**	**know *n* (%)**
Specialized smoking cessation clinics	101 (15.0)	147 (21.8)	68 (10.1)	359 (53.2)
University hospital	50 (7.4)	106 (15.7)	95 (14)	424 (62.8)
On-campus clinic	58 (8.6)	90 (13.3)	108 (16)	419 (62.1)
Family physicians	82 (12.1)	96 (14.2)	75 (11.1)	422 (62.5)

When students' responses were compared on the basis of gender, a significant difference was found with respect to the number of cigarettes smoked per day, history of smoking (*p* = 0.0001), frequency of smoking (*p* = 0.0001), whether a quitting smoking was tried or not (*p* = 0.0001). When similar approach was applied on the basis on age, a significant difference was found based on the history of smoking only (*p* = 0.015). Additionally, education on the danger of smoking was found to be significantly influenced by gender and age. Further information is provided in Table 5.

**Table 5:** The association between smokers, smoking behaviors with respect to age, gender.

**Questionnaire**	**Gender**			**Age (year)**				
	**Male (*n*)**	**Female** **(*n*)**	***P*-value**	**18 to <23** **(*n*)**	**23 to <26 (*n*)**	**26 to <30** **(*n*)**	**30 years (*n*)**	***P-*value**
A number of cigarettes do you smoke per day?^*^^#^								
<10	29	09		17	18	02	01	
10–20	61	07	0.010	42	20	03.	03	0.465
>20	11	02		05	06	02	–	
History of smoking? ^*^^#^								
(a) <1 Year	01	07		07	–	–	01	
(b) 1 to 3 years	25	08	0.0001	25	08	–	–	0.015
(c) >3 years	85	11		43	43	07	03	
Frequency of smoking?^*^^#^								
(a) Daily smoker	98	12		57	43	07	03	
(b) Occasional smoker	12	14	0.0001	17	08	–	–	0.545
(c) Sometimes	01	–		01	–	–	01	
Tried to quit smoking? ^*^^#^								
(a). Yes, more often	21	07		16	08	02	02	
(b). Yes, sometimes	64	03	0.0001	30	34	03	–	0.124
(c). No, I never tried	16	10		18	04	02	02	
(d). I don't want to quit smoking	09	06		10	05	–	–	
Have you received any education on the dangers of smoking^**^?								
(a). Yes	110	104		153	51	07	03	
(b). No	156	120	0.007	193	70	09	04	0.043
(c). I don't know	77	108		138	29	09	09	

** *(N = 675)*;

* *(N = 137)*;

# *Missing response*.

## Discussion

Tobacco smoking represents a major health challenge faced by the National Health Service as it predisposes smokers to several health threats such as cancer and cardiovascular diseases (26). Our study showed that 20.3% of pharmacy students were smokers, which is higher than previous study by Al Arifi M. among pharmacy' students (13.4%) (27) and Alotaibi et al. among Saudi students (17%) (28). However, our study results were substantially lower compared to the previous results reported by Parthasarathi et al. among health care (28.9%) (29). The prevalence of smokers in our participants is mainly and significantly influenced by the age and gender of students. The majority of our pharmacy students had positive and satisfied attitude toward anti-smoking behavior, which resonates with the prevalence of non-smokers (78.7%). This is closely similar to a cross-sectional study conducted by Sychareun et al. and colleagues on a sample of pharmacy students in the University of Health Sciences in Vientiane, Laos, where 78.7% were non- smokers, 1.5% were current smokers and 24.2% were ex-smoker (26).

Our study revealed that roughly one-third of pharmacy students received official education on the hazards of smoking, which was greater than Sychareun et al. study on a pharmacy student (25%) (26) and Parthasarathi and colleagues on a group of pharmacy students in Mysore, India (29.7%) (29). Nevertheless, it was lower compared to medical students in Turkey (48.2%) (30). Most of our pharmacy students (89.8 %) believed that health care professionals should advocate and promote SC via practicing in advising patients about SC that could potentially enhance the chance to quit smoking. Unfortunately, it has been observed that in many developing countries, health care providers seldom discuss this topic with their patients (31, 32). Despite that, the Ministry of Health in Saudi Arabia has worked relentlessly on this issue and started the Tobacco Control Program which provides services such as increasing awareness toward smoking and its harmful effect and promoting the use of SC methods, in series of SC clinics all over the kingdom, to combat smoking habit (33). The impact of this program will increase by having strict smoking-free policies and regulations on tobacco sales and smoking like forbidding tobacco sales to adolescents and banning smoking in public places, which were strongly believed by our students, 92.2 and 80.6%, respectively.

With respect to awareness about the usefulness of SC methods among our participants, most of students were not fully aware of the importance and effectiveness of SC methods despite being heard of them previously. Additionally, the majority believed that education about SC programs should be incorporated into their academic curricula to enhance their knowledge about SC methods. Increased cigarette prices, on the other hand, are another intervention to reduce smoking prevalence in Saudi Arabia and other nations, as indicated by a previous study in Saudi Arabia (34). Few limitations exist in our study. First, the design of research was an online based-cross sectional self-reported survey, potentially rendering our results less reliable. However, because the survey was anonymous and completely voluntary, one can assume that smoking status was reliably captured. Secondly, the study was conducted on a single university's students, hence results cannot be generalized to all pharmacy students in Saudi Arabia. Third, despite assessing the beliefs and attitude toward SC methods, the level of knowledge about SC methods was not evaluated among the students.

## Conclusion

The prevalence of non-smokers among pharmacy students is encouraging, yet level of awareness about and usefulness of SC methods is unsatisfactory. Therefore, the study clearly describes that there is a great need for integrating SC programs in their academic curricula to prepare them for real-world practice.

## Data Availability Statement

The raw data supporting the conclusions of this article will be made available by the authors, without undue reservation.

## Ethics Statement

The studies involving human participants were reviewed and the study was approved by the institutional review board of King Saud University College of Medicine with the following reference number: E-21-6430. The patients/participants provided their written informed consent to participate in this study.

## Author Contributions

AA, MA-A, and SW: conceptualization and methodology. SS and SW: software. AA, SS, and SW: validation. SW and SS: formal analysis. AA and MA-A: investigation and supervision. AA, YS, and IA: data curation. SS, AA, MA-A, and SW: writing – original draft preparation. AA: project administration. All authors reviewed the manuscript. All authors contributed to the article and approved the submitted version.

## Funding

This study is funded by Researchers Supporting Project (Project number RSP-2021/81), King Saud University, Saudi Arabia.

## Conflict of Interest

The authors declare that the research was conducted in the absence of any commercial or financial relationships that could be construed as a potential conflict of interest.

## Publisher's Note

All claims expressed in this article are solely those of the authors and do not necessarily represent those of their affiliated organizations, or those of the publisher, the editors and the reviewers. Any product that may be evaluated in this article, or claim that may be made by its manufacturer, is not guaranteed or endorsed by the publisher.
